# Evidence of non-pancreatic beta cell-dependent roles of Tcf7l2 in the regulation of glucose metabolism in mice

**DOI:** 10.1093/hmg/ddu577

**Published:** 2014-11-14

**Authors:** Kathleen A. Bailey, Daniel Savic, Mark Zielinski, Soo-Young Park, Ling-jia Wang, Piotr Witkowski, Matthew Brady, Manami Hara, Graeme I. Bell, Marcelo A. Nobrega

**Affiliations:** 1 Department of Human Genetics,; 2 Department of Medicine,; 3 Department of Surgery; 4 Department of Medicine, Section of Endocrinology, Diabetes, and Metabolism, University of Chicago, Chicago, IL 60637, USA

## Abstract

Non-coding variation within *TCF7L2* remains the strongest genetic determinant of type 2 diabetes risk in humans. A considerable effort has been placed in understanding the functional roles of TCF7L2 in pancreatic beta cells, despite evidence of *TCF7L2* expression in various peripheral tissues important in glucose homeostasis. Here, we use a humanized mouse model overexpressing *Tcf7l2*, resulting in glucose intolerance, to infer the contribution of *Tcf7l2* overexpression in beta cells and in other tissues to the metabolic phenotypes displayed by these mice. Restoring *Tcf7l2* expression specifically in beta cells to endogenous levels, in face of its overexpression elsewhere, results in impaired insulin secretion, reduced beta cell number and islet area, corroborating data obtained in humans showing similar phenotypes as a result of manipulations leading to Tcf7l2 loss of function. Interestingly, the persistent overexpression of *Tcf7l2* in non-pancreatic tissues results in a significant worsening in glucose tolerance *in vivo*, indicating that *Tcf7l2* overexpression in beta cells does not account for the glucose intolerance in the *Tcf7l2* overexpression mouse model. Collectively, these data posit that Tcf7l2 plays key roles in glucose metabolism through actions beyond pancreatic beta cells, and further points to functionally opposing cell-type specific effects for Tcf7l2 on the maintenance of balanced glucose metabolism, thereby urging a careful examination of its role in non-pancreatic tissues as well as its composite metabolic effects across distinct tissues. Uncovering these roles may lead to new therapeutic targets for type 2 diabetes.

## Introduction

Genetic variation within introns of *TCF7L2* is strongly associated with increased risk of type 2 diabetes (T2D) (1–8). This non-coding region contains *cis*-regulatory elements that drive expression of *TCF7L2* across a variety of tissues involved in glucose homeostasis, suggesting that the risk variants likely alter the expression of *TCF7L2* (9–11). Notably, reports have shown that the most strongly T2D-associated single nucleotide polymorphism (SNP), rs7903146, resides within a region of open chromatin with demonstrated *cis*-regulatory activity in pancreatic beta cells, as well as other tissues (11–13). Furthermore, the T2D-risk (T) allele for this SNP drives stronger enhancer activity than the protective (C) allele (11–13). Together, these data suggest that the T2D-risk variant(s) leads to increased *TCF7L2* expression, corroborating early observations that the T2D-risk allele of rs7903146 is associated with increased *TCF7L2* mRNA expression in pancreas, even in non-diabetic individuals (14).

A link between increased expression of *Tcf7l2* and glucose intolerance phenotypes has been further supported by mouse models. Mice homozygous for a Tcf7l2 null allele are born with dramatically low blood glucose levels and die perinatally partially due to hypoglycemia (9,15,16). Mice heterozygous for germline null Tcf7l2 alleles are viable and grow normally, mirroring the same pattern of reduced blood glucose and plasma insulin levels along with improved glucose tolerance and insulin sensitivity, even after a high fat diet regimen (9,15,16). Conversely, mice that overexpress *Tcf7l2* display reciprocal phenotypes, including increased plasma insulin levels and glucose intolerance due to peripheral insulin resistance, indicating that overexpression of *Tcf7l2* leads to a type 2 diabetic phenotype (9).

Despite these results, a significant body of evidence has accumulated over the past 7 years suggesting that genetic or molecular manipulations leading to *Tcf7l2* loss of function in pancreatic beta cells lead to diabetogenic phenotypes. *In vitro* studies on human and rodent islets have found that silencing of *TCF7L2* leads to decreased proinsulin production and processing, decreased insulin secretion, decreased islet number, decreased beta cell proliferation and increased apoptosis (17–21). Complementary studies suggest increased *Tcf7l2* expression fosters beta cell regeneration (20,22). Collectively, these data suggest that reduced *Tcf7l2* expression leads to beta cell depletion and malfunction, hyperglycemia and T2D-like phenotypes. Further work has recently demonstrated that *Tcf7l2* is a master regulator of insulin production and processing in both rodent and human beta cells (21). This study from Zhou *et al.* identified direct targets of TCF7L2 including ISL1 and the subsequent downstream targets that direct proinsulin production and processing; these molecular targets are perturbed upon TCF7L2 silencing. Mouse *in vivo* experiments also seem to support a role for *Tcf7l2* as a regulator of insulin production and secretion. Recent work from Guy Rutter's laboratory has shown that a beta cell-specific knock-out of Tcf7l2 leads to decreased beta cell volume, decreased insulin secretion, and subsequent glucose intolerance in mice fed high fat diet (23).

Together, these studies have painted a difficult pathophysiological picture to interpret. While human genetics and genomics data suggest that increased T2D risk is associated with increased *TCF7L2* expression, a notion supported by various genetically engineered mouse models, other studies focused on the beta cell roles of TCF7L2 have pointed in the opposite direction, suggesting that a *TCF7L2* loss of function in beta cells may represent the causal link between genetic variation in the TCF7L2 locus and T2D risk. To clarify these conflicting functional results, we sought to determine the relative contribution of *Tcf7l2* in beta cells to the T2D phenotypes seen in mice overexpressing *Tcf7l2*. Toward that, we engineered mice with *Tcf7l2* expression restored to endogenous baseline levels in beta cells while maintaining overexpression in peripheral metabolic tissues where *Tcf7l2* is normally expressed, including brain, liver, gut and fat.

Our data show that reducing *Tcf7l2* expression from high to normal levels in adult mouse beta cells results in blunted insulin secretion, reduced plasma insulin levels and selective loss of pancreatic beta cells, corroborating data from *in vitro* studies. Importantly, our results demonstrate that in face of this restored pancreatic expression of *Tcf7l2*, overexpression in other tissues still maintains the glucose intolerance phenotypes, including further amplifying these metabolic perturbations, strongly suggestive of important roles for *Tcf7l2* in regulation of glucose metabolism beyond its well-established roles in beta cells.

## Results

### Inducible excision of transgenic *Tcf7l2* in beta cells

We utilized a mouse model of *Tcf7l2* overexpression that we have previously described (9). These mice have been engineered to carry a human BAC corresponding to the *TCF7L2* locus that further spans the 92 kb T2D-associated region. One copy of the mouse full-length cDNA was recombineered into the TCF7L2 translation start site within the BAC. Three copies of the BAC integrated randomly into the genome. Consequently, mice carrying these three BACs express transgenic mouse *Tcf7l2* regulated by human *cis*-regulatory elements, in addition to the expression of the two endogenous copies of *Tcf7l2* in the mouse genome. This results in a humanized model of overexpression, where *Tcf7l2* is overexpressed at moderate levels in physiological tissues, determined by the endogenous regulatory landscape of the *TCF7L2* human locus. This result in a 50–200% increase in the expression level of *Tcf7l2* in transgenic mice compared with wild-type (9). Importantly, the *Tcf7l2* cDNA recombineered in the human BAC is floxed, allowing for the conditional removal of the transgenic *Tcf7l2* copies via Cre-recombination, and thereby restoring normal *Tcf7l2* expression level in a tissue-specific manner. We favored this model of conditional gene excision over the routine conditional knock-out strategies because Tcf7l2 is a transcription factor with well-established developmental roles. Indeed, a conditional ablation of a transcription factor may lead to altered cell specification, proliferation, etc., which may confound the adult onset glucose metabolism phenotypes we are interested in.

**Figure 1.**
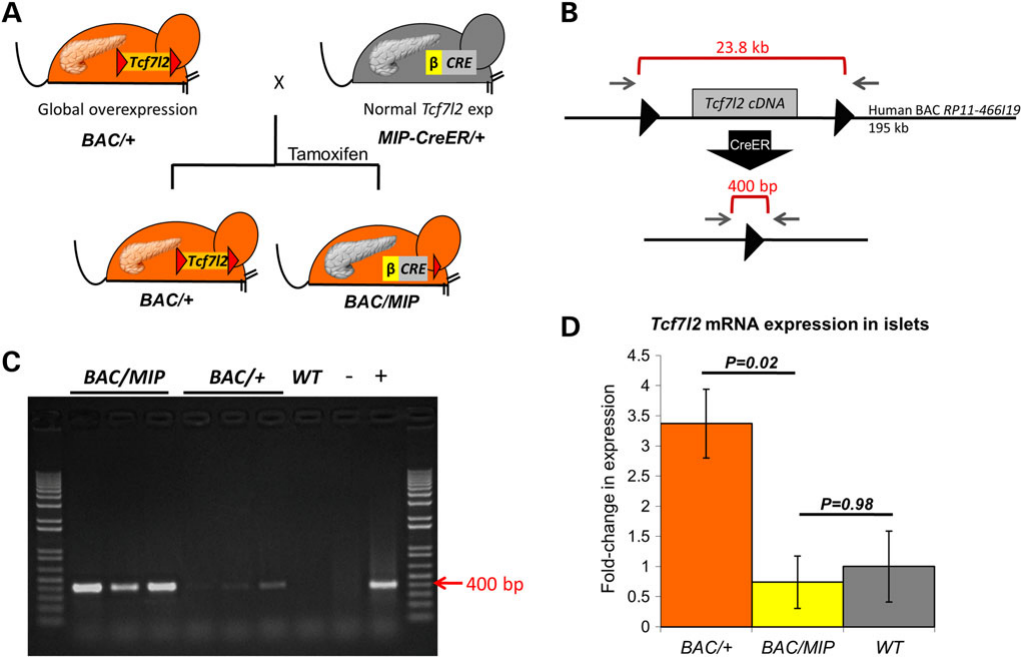
Beta cell-specific ablation of *Tcf7l2* cDNA and restoration of *Tcf7l2* expression. (**A**) Breeding scheme to create *BAC/MIP* mice. Hemizygous *BAC/+* mice (orange) are shown carrying the *Tcf7l2* cDNA in the BAC bordered by loxP sites (red triangles) and a pancreas with overexpression of *Tcf7l2* (orange). Hemizygous *MIP-Cre/+* mice (gray) are shown carrying the beta cell-specific MIP-Cre (gray) a pancreas with normal expression of *Tcf7l2* (gray). Crossing these mice creates F1 offspring inheriting the BAC with no Cre (*BAC/+*) with continued overexpression of *Tcf7l2*, globally and including the pancreas (orange). This cross also produces mice that inherit both the BAC and the MIP-Cre, which upon activation by tamoxifen, cleaves at loxP sites to restore normal expression of *Tcf7l2* in beta cells only (gray pancreas) while maintaining overexpression elsewhere (orange remainder of mouse). (**B**) Diagram of human BAC containing mouse Tcf7l2 cDNA floxed by loxP sites (black triangles). Gray arrows indicate PCR primers which can only amplify the 400 bp fragment after excision by Cre. (**C**) PCR amplification of 400 bp fragment in islets after excision by MIP-Cre. (D) Expression of *Tcf7l2* in *BAC/+, BAC/MIP* and wild-type islets quantified by qRT-PCR.

To selectively ablate *Tcf7l2* overexpression in beta cells, we utilized an inducible beta cell-specific mouse insulin promoter-1 CreER (*MIP-CreER*) allele, previously described (24). Importantly, this CreER driver lacks hypothalamic Cre expression that other insulin promoter Cre have reported. We crossed the *MIP-CreER* mice to our *Tcf7l2*-BAC transgenic mice, creating F1 mice that are both BAC positive and MIP-CreER positive (*BAC/MIP*, Fig. 1A). Since the MIP-CreER is inducible, we injected *BAC/MIP-CreER* mice with tamoxifen to activate Cre, resulting in an adult-specific ablation of the *Tcf7l2* transgenic copies in beta cells. Mice that do not inherit the MIP-CreER maintain global overexpression and are therefore referred to as *BAC/+* henceforth (Fig. 1A).

To assay for the efficiency of CreER-mediated excision of the floxed 24 kb region of the BAC harboring the *Tcf7l2* cDNA in *BAC/MIP* mice, we used primers outside the loxP sites to amplify the resulting 400 bp segment after CreER excision (Fig. 1B). As expected, PCR on islet DNA from *BAC/MIP* mice amplified the segment confirming an activated Cre and the subsequent removal of the floxed region containing the extra copies of *Tcf7l2* cDNA (Fig. 1C).

We next assayed the impact of the excision of the transgenic *Tcf7l2* copies in beta cells at the transcription level. We isolated mRNA from both *BAC/+* and *BAC/MIP* islets and performed quantitative reverse transcription PCR (qRT-PCR) to determine whether removal of *Tcf7l2* cDNA from the BAC subsequently reduced *Tcf7l2* expression in *BAC/MIP* islets. We observed overexpression of *Tcf7l2* in *BAC/+* islets matching the expression levels seen in the pancreas of the founder global overexpression mouse (9). Reflecting the excision of the transgenic copies of *Tcf7l2* in *BAC/MIP* mice, we detected a significant decrease in *Tcf7l2* expression in *BAC/MIP* islets, which now express *Tcf7l2* at the same level as wild-type islets (Fig. 1D). These results illustrate our ability to generate a mouse model where we can ablate *Tcf7l2* overexpression specifically in beta cells of adult mice, while preserving overexpression in peripheral tissues.

### Glucose intolerance in *Tcf7l2* overexpression mice

*Tcf7l2* overexpression in *BAC/+* mice results in increased insulin secretion and glucose intolerance after high fat diet (HFD) due to peripheral insulin resistance (9). Therefore, we assayed *BAC/MIP* mice for changes in glucose metabolism and insulin secretion to identify the specific contribution of *Tcf7l2* overexpression in beta cell function.

**Figure 2.**
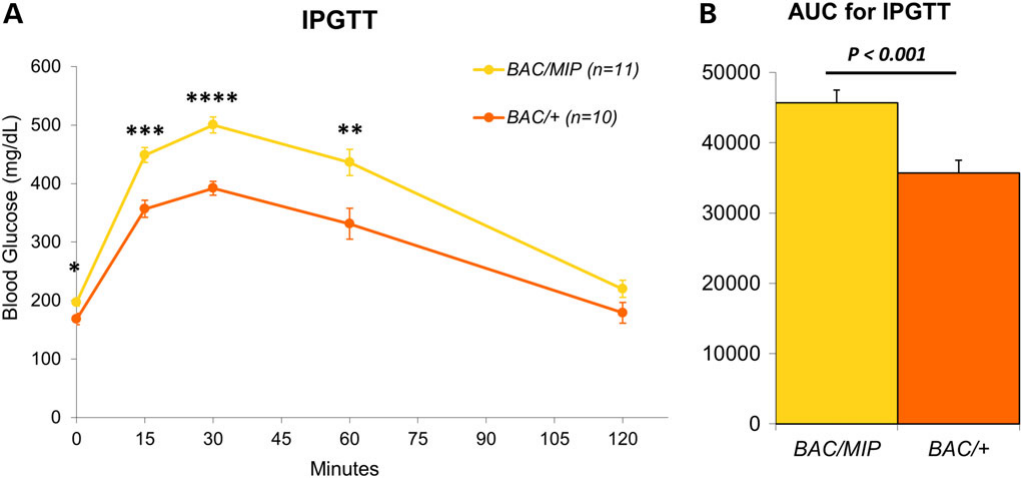
Glucose tolerance of *BAC/MIP* mice. (**A**) IPGTT for *BAC/+* (orange) and *BAC/MIP* (yellow) mice after 10 weeks high fat diet. Injected 2 g/kg dextrose. (**B**) AUC of the IPGTT plot from (A) with *P* values as shown. *P < 0.05; **P < 0.01; ***P < 0.001; ****P < 0.0001.

We subjected both *BAC/MIP* and *BAC/+* mice to HFD for 10 weeks. During this time, we tracked mouse weight gain and found no difference between BAC/MIP and BAC/+ mice. After HFD feeding, we performed an intraperitoneal glucose tolerance test (IPGTT). We observed that restoring wild-type levels of *Tcf7l2* expression in the beta cells of *BAC/MIP* mice resulted in a significant worsening of glucose tolerance compared with *BAC/+* littermates (Fig. 2A and B). We replicated this observation in a second cohort of mice, including *BAC/+*, *BAC/MIP* and wild-type littermates, where we injected half the amount of glucose during IPGTT. Wild-type mice displayed the strongest glucose tolerance among the three groups after 10 weeks of HFD. *BAC/+* mice were glucose intolerant and *BAC/MIP* mice displayed the most severe glucose intolerance of the three groups, even with the attenuated glucose injection, confirming our earlier results (Supplementary Material, Fig. S1A and B). These results suggest that *Tcf7l2* overexpression in beta cells of *BAC/+* mice does not represent the main contributor to the glucose intolerance seen in these mice, stressing the role of extra-pancreatic tissues in the Tcf7l2-mediated glucose intolerance.

**Figure 3.**
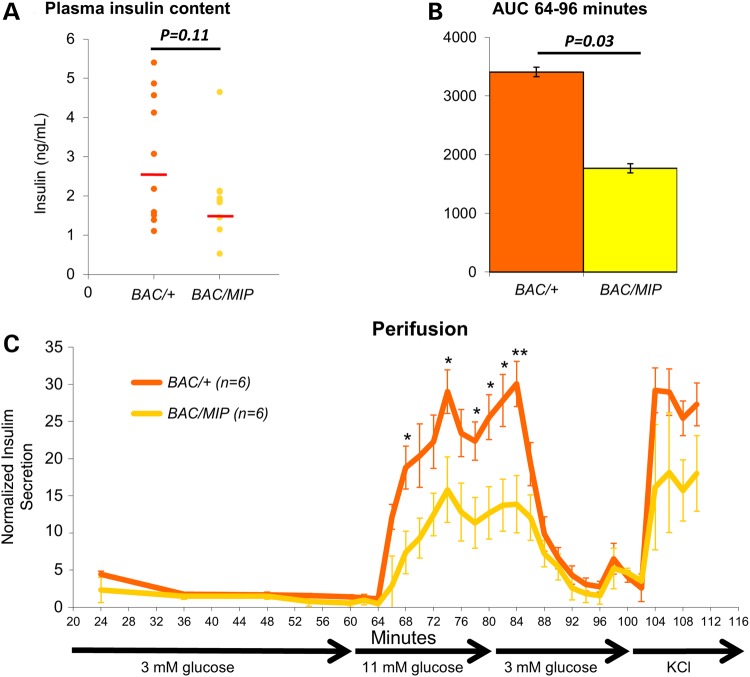
Beta cell analysis of insulin content and secretion. (**A**) Fasting plasma insulin levels in *BAC/+* (orange) and *BAC/MIP* (yellow) mice after 10 weeks high fat diet (HFD). (**B**) AUC of insulin secretion measured by ELISA during insulin spike from 64 to 96 min of perifusion. (**C**) Normalized insulin levels secreted by isolated islets during glucose perifusion. Minutes indicate time since initial incubation of islets in glucose. Islets were incubated in varying glucose concentrations; 3 mm glucose from 0 to 60 min, 11 mm glucose from 60 to 80 min, 3 mm glucose from 80 to 100 min, and final incubation in KCl from 100 to 110 min. Insulin samples were collected every 12 min from 24 to 60 min and then every 2 min for the remainder. Insulin secretion from islets was quantified by insulin ELISA. *P < 0.05; **P < 0.01.

Next, we determined if the reduced *Tcf7l2* expression in beta cells and the subsequent worsening of glucose intolerance are coupled with changes in plasma insulin content. Using an insulin enzyme-linked immunosorbent assay (ELISA), we observed *BAC/MIP* mice to have noticeably reduced circulating insulin compared with *BAC/+* mice (Fig. 3A). This reduction of plasma insulin levels in *BAC/MIP* mice coincides with the worsening of glucose tolerance, suggesting this is the main mechanism by which glucose tolerance is worsened in *BAC/MIP* mice. This reduced plasma insulin content may further reflect impaired insulin secretion from islets. We subsequently assayed insulin secretion directly from *BAC/+* and *BAC/MIP* islets in response to glucose stimulation using islet perifusion (25–27). When exposed to a concentrated glucose solution, *BAC/MIP* islets secrete significantly less insulin than *BAC/+* islets (Fig. 3B and C), indicating impaired insulin secretion secondary to a reduction in *Tcf7l2* expression in beta cells. Together, these results demonstrate a dose-dependent curve of insulin secretion related to *Tcf7l2* expression levels, mimicking the blunted insulin secretion response described as a result of reduced *Tcf7l2* expression in beta cells in humans and mice (17–21).

### Reduced beta cell area in *BAC/MIP* mice

**Figure 4.**
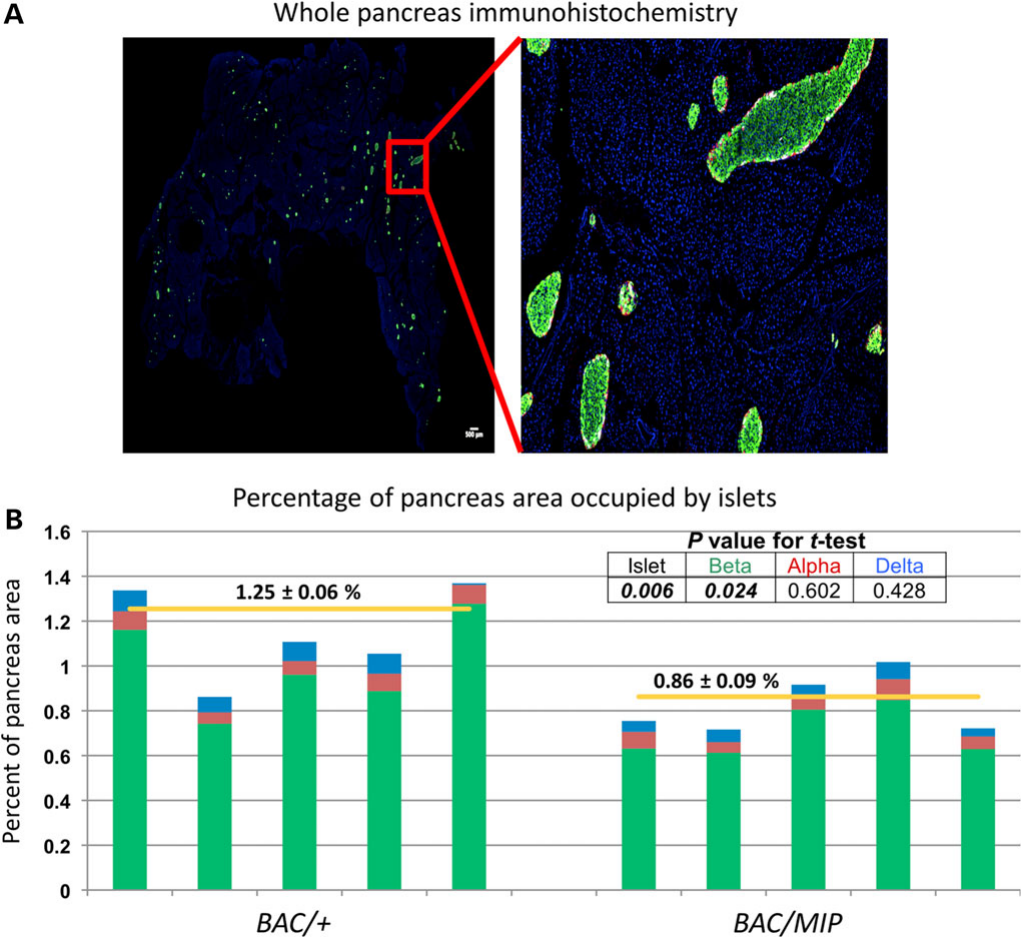
Quantification of beta cell area. (**A**) Representative image of immunohistochemistry of whole-mouse pancreas with beta cells (green), alpha cells (red) and delta cells (white) and nuclei (blue). (**B**) Quantification of islet composition and area. The total endocrine cell area is normalized to the total pancreas area in each mouse; *BAC/+ n* = 5, *BAC/MIP n* = 5. Total islet area is then subdivided into its cellular components; beta cell area in green, alpha cell area in red, delta-cell area in blue. Yellow horizontal lines indicate the average total islet area for each group. *BAC/+* and *BAC/MIP* mice are compared at total islet area as well as area of each cell type, with *P* values reported in table.

The blunted insulin secretion in *BAC/MIP* mice could be reflective of a decrease in beta cell number supported by recent data reporting a preferential loss of beta cells in patients with T2D (28). To assess this possibility, we utilized immunohistochemistry on whole pancreas from age- and weight-matched *BAC/+* and *BAC/MIP* mice using antibodies against insulin, glucagon and somatostatin, as well as DAPI nuclear staining (Fig. 4A). Using a large-scale computer-assisted imaging method, we quantified endocrine cell mass and islet cellular composition. This study showed that a reduction of *Tcf7l2* expression in *BAC/MIP* islets leads to a significant decrease in beta cell and total islet area (*P* < 0.006, two-sided Student's *t*-test) compared with *BAC/+* overexpression mice (Fig. 4B). Importantly, we observed no decrease in alpha or delta cell area in *BAC/MIP* islets (*P* = 0.602 and 0.428, respectively, two-sided Student's *t*-test), indicating that our ablation of *Tcf7l2* overexpression has a localized effect specifically in beta cells (Fig. 4B). These data further indicate that beta cells with less *Tcf7l2* expression occupy less pancreatic area, supporting previous evidence that *Tcf7l2* expression is important for beta cell survival and proliferation (17,18,20,22).

## Discussion

Considerable efforts from various research groups, including our own, have highlighted a key role for Wnt-signaling in the regulation of energy metabolism, and these analyses have eluded to potential mechanisms of how misregulation of this conserved developmental pathway leads to increased susceptibility to T2D (29–31). However, most of these studies have largely concentrated on the roles of TCF7L2 in pancreatic beta cell. This limited focus was initially precipitated by the demonstration that reducing Tcf7l2 expression in rodent and human beta cells models can lead to impaired insulin secretion and production (17,18). Human genetics data have added further support by demonstrating that the T2D-risk allele is associated with impaired insulin secretion in humans, even in non-diabetics (32).

Nevertheless, the concerted effort to understand the link between beta cell *TCF7L2* expression and T2D susceptibility has often met with contradictory results. At the heart of the contradiction is the direction of change in *TCF7L2* expression leading to glucose intolerance and T2D susceptibility. Studies in human islets agree the T2D-risk allele is associated with increased *TCF7L2* expression and decreased insulin secretion (12–14,33). However, *in vitro* analyses on isolated human and rodent islets suggest decreased *TCF7L2* expression is associated with decreased insulin secretion (17–19,21,34). These studies also highlight other islet defects following *TCF7L2* downregulation, including fewer GLP-1 and GIP receptors, decreased expression of genes involved in insulin vesicle fusion, and increased beta cell apoptosis (17–19,21). These results suggest decreased *TCF7L2* expression drives beta cell malfunction, decreased insulin secretion and decreased glucose tolerance, but paradoxically the risk allele remains strongly associated with increased *TCF7L2* expression.

Mouse models harboring both gain and loss of function in Tcf7l2 do not readily reconcile these differences. Mice overexpressing *Tcf7l2* either in multiple tissues or specifically in the liver display impaired glucose tolerance (9,15). While germline null *Tcf7l2* alleles have repeatedly been shown to result in improved glucose tolerance, beta cell-specific ablation of *Tcf7l2* in mice often leads to the opposite phenotype, a worsening in glucose tolerance (9,16,23,35) or to no change in glucose tolerance at all (15). These data seem to be at odds with the observation that, in humans, the T2D-associated SNP within TCF7L2 is associated with impaired insulin secretion. Further studies will determine whether this is a fundamental biological difference between humans and mice.

In hopes of resolving these incongruencies, various explanations have been raised. Chief among those is the idea that perhaps mice do not represent a viable model of TCF7L2 biology in humans, a concept that has recently been demonstrated in connection to other genes involved in T2D susceptibility emerging from Genome-Wide Association Studies (36,37).

Our study helps to reconcile some of the conflicting data on the beta cell contribution to the *Tcf7l2* glucose intolerance phenotype. We demonstrate that overexpression of *Tcf7l2* in the multiple tissues where it is normally expressed leads to glucose intolerance, but reducing *Tcf7l2* expression to wild-type levels in beta cells while maintaining overexpression elsewhere increases the severity of the glucose intolerance phenotype. Furthermore, this reduction of expression in beta cells is accompanied with decreases in insulin secretion and a reduction in beta cell area and numbers. These phenotypes are precisely those that have been systematically shown in human and mouse cell lines (17–19,21,34), suggesting that the biology of TCF7L2 as a central regulator of glucose metabolism is conserved between human and mouse. We posit that part of the seemingly contradictory data generated over the past several years is due to the evaluation of TCF7L2 impact on beta cells in an ex-vivo context. Specifically, we do see the same phenotypes associated with Tcf7l2 in beta cells, but within a whole-system mouse physiological context, *Tcf7l2* is also expressed in multiple other tissues relevant to glucose homeostasis (38), and it seems that its roles in one or more of these peripheral tissues overrides the pancreatic actions of this factor, highlighting intricate higher-order multi-organ system interactions that can be faithfully observed within a complex multicellular experimental model.

Other studies support the conclusion that *Tcf7l2* overexpression in the periphery leads to glucose intolerance. The T2D-associated genomic interval has been shown to be a complex regulatory region driving *TCF7L2* expression in a variety of peripheral tissues, with the risk allele maintaining allele-specific effects (9–11). Recent data from Hans Clevers' laboratory indicate that *TCF7L2* is an important regulator of liver metabolism and gluconeogenesis; liver-specific *Tcf7l2* knock-out mice demonstrate decreased gluconeogenesis and improved glucose tolerance while transient overexpression of *Tcf7l2* leads to increased gluconeogenesis (15).

Our data are also congruent with that from the Rutter laboratory, showing that reduced *Tcf7l2* expression in beta cells *in vivo* leads to decreased beta cell volume, decreased glucose and Glp-1 stimulated insulin release and decreased glucose tolerance compared with wild-type (23,35). We observe similar phenotypes when we reduce *Tcf7l2* expression from high to normal levels in our mice. In line with a worsening of glucose tolerance in our mouse models, we would predict that a beta cell-specific *Tcf7l2* loss of function would lead to the same phenotypes described by Rutter and colleagues.

Our study highlights the complexities of whole-systems *in vivo* physiology, where multiple organs and tissues regulate the same biological function, often with opposing effects (39). Our data point to a classical example where manipulating a single gene, in this case *Tcf7l2*, may lead to a phenotype in one tissue, even when the overall *in vivo* phenotypic impact of that gene may be in direct opposition. Importantly, our work emphasizes the pressing need to better characterize the roles of TCF7L2 in other peripheral tissues. Even supposing the genetic association with T2D is demonstrated to arise from TCF7L2’s function in pancreatic beta cells, these data provide evidence that the role of TCF7L2 in regulating glucose metabolism from other tissues seems to be more robust. Harnessing the biology of TCF7L2 in these additional tissues may hold the key to developing novel and better therapeutic targets for T2D.

## Materials and Methods

### Animals

We used the CD1 *Tcf7l2* overexpression mouse harboring the human BAC RP11–466I19 containing the full-length mouse *Tcf7l2* cDNA (GenBank BC052022.1) as previously described (9). This BAC spans the entire T2D-associated LD block in humans, including all *cis*-regulatory elements within it. This results in a mouse that overexpresses Tcf7l2 only in tissues endogenously expressing this gene in humans. Hemizygous mice (*BAC/+)* contain three extra copies of floxed mouse *Tcf7l2* cDNA leading to global *Tcf7l2* overexpression (9). Hemizygous BAC mice were crossed to C57B/6 mice hemizygous for the inducible mouse insulin promoter Cre, *MIP-CreER*, as previously described (24). This cross of *BAC/+* to *MIP/+* generates *BAC/+*, *MIP/+* wild-type (*WT*) and *BAC/MIP-CreER* F1 males. *MIP/+* were excluded from further study. *BAC/MIP-CreER* males were injected with 200 µL of 10 mg/mL tamoxifen for 3 days at 5–7 weeks of age to activate the MIP-CreER. Active MIP-CreER in beta cells leads to excision of the floxed region of the BAC containing the extra copies of Tcf7l2 cDNA.

### Verification of islet-specific conditional ablation of the transgenic Tcf7l2 copies

To verify active MIP-CreER and the subsequent excision of the floxed region in the BAC, we performed PCR on islet DNA using primers outside of the floxed region. Since the entire floxed region of the BAC is 23.8 kb, no excision of the region will prevent amplification. Proper excision of the floxed region results in a 405 bp fragment, easily amplified by PCR. Islets were isolated from 3 *BAC/+*, 3 *WT* and 3 *BAC/MIP* mice as previously described (40) and DNA was isolated using the Protein Precipitation Solution (Qiagen). Standard PCR conditions were used and the primer sequences are as follows: F—GTTTCTGGGTGAGCAAAAACA, R—CGTGAGACTACGATTCCATCAAT.

While this PCR technique validates CreER excision of the floxed region at the DNA level, we also verified removal of the overexpression at the RNA level. Islets isolated from 3 *BAC/+*, 3 *WT* and 3 *BAC/MIP* mice were used for RNA extraction using Tri-Reagent (Sigma) and then for cDNA generation using Superscript II reverse transcriptase (Invitrogen). *Tcf7l2* exonic primers (F—ATCGTCACACCGACAGTCAA, R—TTGGAGTCCTGATGCTTTGAG) and *Hprt* housekeeping gene primers (F—TGTTGTTGGATATGCCCTTG, R – GCGCTCATCTTAGGCTTTGT) were used for qRT-PCR.

### In vivo glucose tolerance experiments

Starting at 5–7 weeks of age, *BAC/+* and *BAC/MIP* mice subsequent to tamoxifen injection were fed a high fat diet (55% fat, Harlan Teklad) for 10 weeks. After this 10-week period, *BAC/+* and *BAC/MIP* mice were tested for glucose sensitivity by IPGTT. Prior to IPGTT, mice were fasted for 4 h and an initial blood glucose reading was taken. This fast was followed by intraperitoneal injection with 2 mg/kg dextrose, and subsequent blood glucose checks using an AccuChek Aviva glucometer (Roche). Blood glucose readings were taken at 15, 30, 60 and 120 min post dextrose injection. After IPGTT, mice resumed high fat diet. This experiment was later repeated including *WT* littermates.

### Blood plasma collection and insulin quantification

After allowing the mice to restabilize for 3–7 days after IPGTT, we again fasted *BAC/+* and *BAC/MIP* mice for 4 hours for blood collection from the tail vein. We collected 500 µl of blood in EDTA coated tubes followed by a 10-min centrifugation at 7000 × g at 4°C for plasma isolation. Plasma was diluted for insulin enzyme-linked immunosorbent assay (ELISA from Millipore) to determine plasma insulin content. *BAC/+*, *BAC/MIP* and *WT* mice were then sacrificed and whole pancreas was collected and collagenase digested to isolate the total pancreatic insulin content. Total pancreatic insulin content from 10 mice per genotype was quantified by insulin ELISA.

### Dynamic islet perifusion

Islets were isolated from six high fat diet fed *BAC/+*and *BAC/MIP* mice for perifusion, which allows for dynamic assessment of islet function based on insulin secretion in response to changing concentrations of glucose (26–28). Islets were incubated in 3 mm glucose for 60 min with insulin samples collected every 12 min. At 60 min, glucose concentration was increased to 11 mm for 20 min and insulin samples were collected every 2 min. Then glucose concentration was returned to 3 mm for 20 min with continued sampling every 2 min. At the end of this 100 min time frame, islets were perifused with KCl for 10 min to release the intracellular insulin. All insulin samples were assessed using ELISA as before and were normalized to the lowest value of basal insulin release during first 60-min incubation in 3 mm glucose. Two insulin secretion peaks upon glucose stimulation are expected, representing current insulin stores and subsequent insulin production. Area under the curve (AUC) is measured from 64–96 min to capture the entirety of the glucose-stimulated insulin response curve.

### Pancreatic histology and cellularity

Five *BAC/+* and 5 *BAC/MIP* mice were sacrificed after 10 weeks high fat diet. Whole pancreas from each animal was dissected and fixed in 4% paraformaldehyde. Paraffin-embedded sections (5 µm) were stained with the following primary antibodies (all 1:500): polyclonal guinea pig anti-porcine insulin (DAKO, Carpinteria, CA), mouse monoclonal anti-human glucagon (Sigma-Aldrich, St. Louis, MO), polyclonal goat anti-somatostatin (Santa Cruz, Santa Cruz, CA) and DAPI (Invitrogen, Carlsbad, CA). The primary antibodies were detected using a combination of DyLight 488, 549 and 649-conjugated secondary antibodies (1:200, Jackson ImmunoResearch Laboratory, West Grove, PA).

Microscopic images were taken with an Olympus IX8 DSU spinning disk confocal microscope (Melville, NY) with imaging software StereoInvestigator (SI, MicroBrightField, Williston, VT). A modified method of ‘virtual slice capture’ was used (41,42). Briefly, the SI controls a *XYZ*-motorized stage and acquires consecutive images, which creates a high-resolution montage composed of images obtained from multiple microscopic fields of view. The entire tissue section was captured as ‘a virtual slice’ using a 10× objective. Each virtual slice taken at four fluorescent channels were further merged into one composite. Given this unique, unbiased, computer-assisted method of ‘virtual slice capture’ one section per pancreas is sufficient to provide representative pancreas composition. Quantification of cellular composition (i.e. each area of beta-, alpha- and delta-cell populations, and islet area by automated contouring of each islet) was carried out using a macro custom-written for Fiji/ImageJ (http://rsbweb.nih.gov/ij/). MATLAB (MathWorks, Natick, MA) was used for mathematical analyses.

### Statistical analyses

Data are shown as the standard error of the mean (SEM). An unpaired two-sided Student's *t*-test was used to test for significance in all cases.

## Supplementary material

Supplementary Material is available at *HMG* online.

## Funding

This work was supported by the National Institute of Health (Grants DK093972, HL123857, HL118758 to M.A.N; DK020595 to G.I.B) and a gift from the Kovler Family Foundation (MH). Funding to pay the Open Access publication charges for this article was provided by Discretionary funds provided to M.A.N. by the University of Chicago.

## Supplementary Material

Supplementary Data
