# Supplementary material for: Evidence of non-pancreatic beta cell-dependent roles of Tcf7l2 in the regulation of glucose metabolism in mice
Source: Hum Mol Genet. 2014 Nov 14;24(6):1646–54. doi: 10.1093/hmg/ddu577 (PMC4381752; doi:10.1093/hmg/ddu577)
Supplement: Supplementary Data [file supp_24_6_1646__index.html]

Evidence of non-pancreatic beta cell-dependent roles of Tcf7l2 in the regulation of glucose metabolism in mice — Evidence of non-pancreatic beta cell-dependent roles of Tcf7l2 in the regulation of glucose metabolism in mice — Supplementary Data 

# Evidence of non-pancreatic beta cell-dependent roles of Tcf7l2 in the regulation of glucose metabolism in mice

## Supplementary Data

Supplementary Data

**Files in this Data Supplement:**

- Supplementary Data - Docx file
