# Supplementary material for: Evidence of non-pancreatic beta cell-dependent roles of Tcf7l2 in the regulation of glucose metabolism in mice
Source: Hum Mol Genet. 2014 Nov 14;24(6):1646–54. doi: 10.1093/hmg/ddu577 (PMC4381752; doi:10.1093/hmg/ddu577)

Supplemental Figure 1. Glucose tolerance of *BAC/+, BAC/MIP,* and *WT* mice. (A) Intraperitoneal glucose tolerance test (IPGTT) for *BAC/+* (orange), *BAC/MIP* (yellow), and *WT* (gray) mice after a ten week high fat diet. Injected 1g/kg dextrose. (B) Area under the curve (AUC) of the IPGTT plot from (A) with *P* values as shown. (*) P < 0.05; (**) P < 0.01.


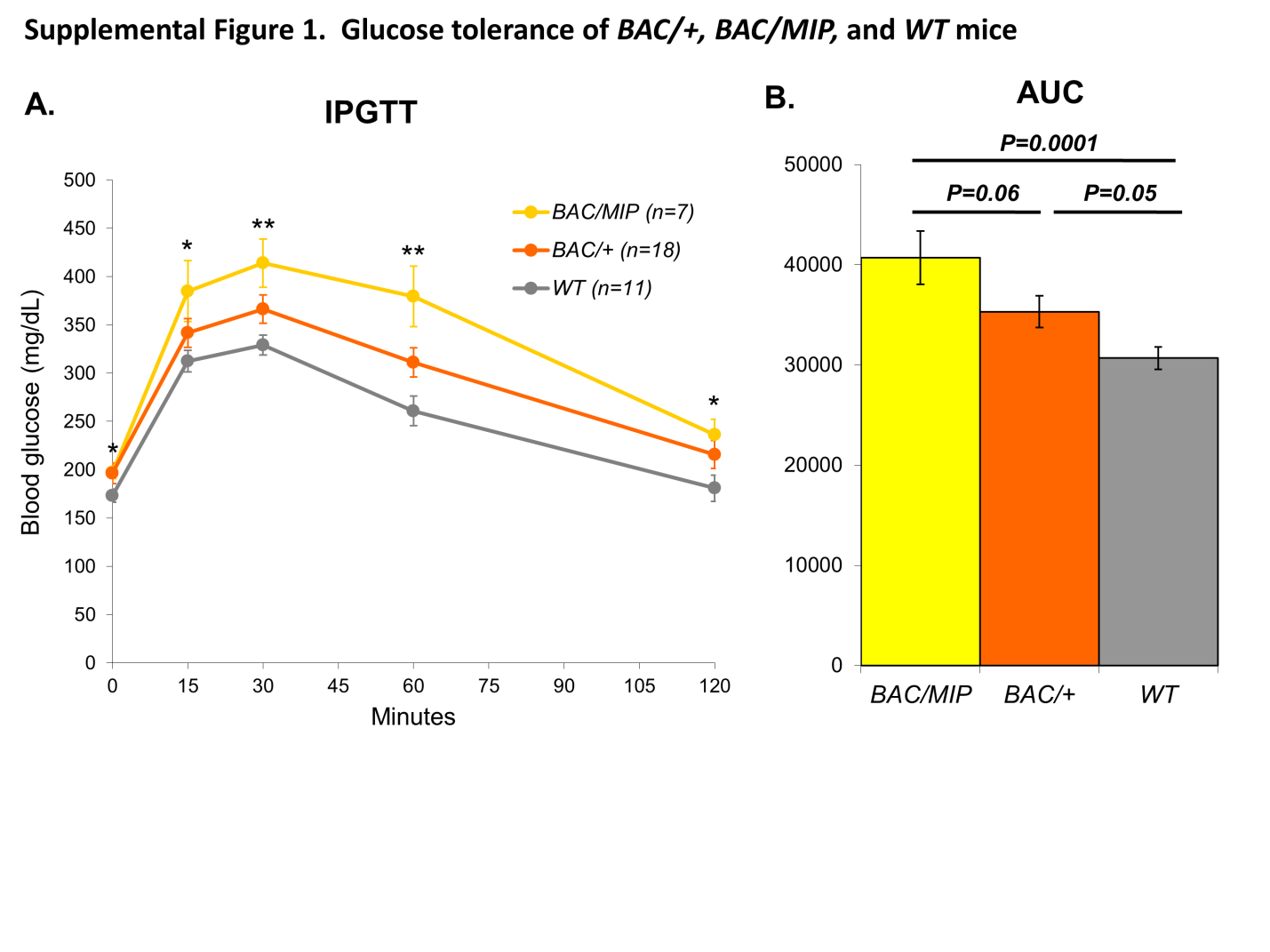

Supplement: Supplementary Data [file supp_ddu577_ddu577supp.docx]
